# The degree and direction of subgenome expression bias in *Brassica napus* vary between cultivars, tissues and developmental stages

**DOI:** 10.1111/tpj.71145

**Published:** 2026-10-10

**Authors:** Hugh Woolfenden, Rachel Wells, Richard J. Morris

**Affiliations:** ^1^ Computational and Systems Biology, John Innes Centre Norwich Research Park Norwich NR4 7UH UK; ^2^ Crop Genetics, John Innes Centre Norwich Research Park Norwich NR4 7UH UK

**Keywords:** homoeologue expression bias, subgenome dominance, *Brassica napus*, oilseed rape, polyploid, transcriptomics, time‐series

## Abstract

Most extant plant lineages show evidence of past polyploidisation events in their genomes. Allopolyploids arise from hybridisation, resulting in polyploid genomes comprising subgenomes from different ancestors. Adaptation to their environment, pre‐ and post‐hybridisation and selection for specific traits has led to several allopolyploids exhibiting an unequal contribution from their subgenomes to phenotypic traits. Associated regulatory changes may have given rise to cultivar‐specific subgenome dominance that contributes to the diversity of phenotypes. Likewise, different tissues and developmental stages have distinct expression profiles that may exhibit higher contributions from one subgenome relative to the other. Here, we investigate different metrics for quantifying the contribution of each subgenome to expression in cultivars of *Brassica napus,* in space (tissue) and time (development). *Brassica napus* inherited two subgenomes, A and C, from its ancestors *Brassica rapa* (A) and *Brassica oleracea* (C). We find that the C subgenome has a larger contribution to transcript abundance than the A subgenome, whereas the mean abundance per expressed gene is higher for the A subgenome. Direct comparison of homoeologous pairs reveals higher expression of genes on the C subgenome. We find that the degree and direction of expression bias can change substantially within a homoeologous pair between cultivars, tissues and across development, suggesting that subgenome dominance is highly dynamic. The quantification of expression bias reveals a strong dependence on the metric and on classification thresholds. These findings help explain contradictory reports on expression bias and subgenome dominance.

## Significance Statement

We show that subgenome dominance in *Brassica napus* is fluid and context‐dependent, varying with genotype, tissue and developmental stage. Quantification reveals that the overall expression bias between the A and C subgenomes of *B. napus* is modest, whilst it can vary dramatically within a homoeologous pair, highlighting that different contexts and different metrics can lead to conflicting inferences.

## INTRODUCTION

Polyploidy is common across the tree of life, arising from whole genome duplication and/or hybridisation events (Morris et al., 2024). Whole genome duplication gives rise to autopolyploidy, that is, multiple copies of the same genome, whereas allopolyploidy refers to the result of a hybridisation event between two different species and the consequent merger of the parental genomes, now termed subgenomes of the new species (Glover et al., 2016). Many important crops are allopolyploids, including several species of *Brassica* (oilseed rape, brown mustard), *Gossypium* (cotton), *Triticum* (wheat) and *Fragaria* (strawberry).

Studies using resynthesised polyploids (Bird et al., 2021; Li et al., 2020; Rapp et al., 2009; Wu et al., 2018) have shown that when different sets of parental chromosomes come together, they undergo a phase of rapid change in which the subgenomes adapt to their new configuration (Alger & Edger, 2020). This dramatic adjustment has been described as a ‘transcriptome shock’ (Hegarty et al., 2006), involving genomic restructuring and epigenetic modifications (Sobhanian et al., 2026).

Previous research using resynthesised lines has revealed that polyploidisation events are followed by structural rearrangements of the genome, silencing of transposons and epigenetic modifications (Wang et al., 2025). Early consequences of these polyploidisation‐induced events include changes in chromatin accessibility that impact gene expression (Fang et al., 2024). Over longer timescales, accumulated mutations can result in the loss of genes from one or more of the subgenomes, termed fractionation, with potentially including the loss of whole chromosomes (Sankoff & Zheng, 2012; Xiong et al., 2011). The genetic redundancy created by polyploidisation leads to genes that have been duplicated from an ancestral single gene copy being under less selection pressure, allowing for sub‐ and neo‐functionalisation (Conant & Wolfe, 2008). These processes are thought to be major drivers of innovation and adaptation to extreme changes in the environment (Sobhanian et al., 2026). Likewise, selective breeding exploits the genetic redundancy of polyploids, resulting in changes in regulation that are tailored towards specific traits and climatic conditions. As a consequence of regulatory adjustments, genes from one subgenome may become more highly expressed than the other(s) and genes inherited from the progenitors may not be lost equally across the subgenomes as the species evolves (Grover et al., 2012). These cases have been described as one subgenome being more ‘dominant’ (Alger & Edger, 2020). Over evolutionary time, polyploids typically revert back to a diploid state (diploidisation) (Bird et al., 2018; Conant et al., 2014), often leaving ancestral remnants of polyploidy that can still be detected (Jiao et al., 2011).

Due to its agronomic importance and interesting history of polyploidisation events, the allotetraploid *Brassica napus* (oilseed rape, OSR) has been the focus of several studies on subgenome dominance. *Brassica napus* is the result of a hybridisation event between its progenitor species, *B. oleracea* and *B. rapa*, about 7500 years ago (Chalhoub et al., 2014). Using a resynthesised line, Wu et al. (2018) examined homoeologue expression in leaves and found a bias to the A subgenome. In a later study that expanded the range of tissues to stems, leaves, flowers and siliques, the A subgenome was found to be dominant in 78.1% of the homoeologous pairs but in some tissues, notably in the leaves and flowers, they identified an expression bias towards the C subgenome (Li et al., 2020). Possible explanations for the apparent discrepancy were discussed and included the leaf age at the time of sampling and the sampled variety (Li et al., 2020). In a separate study in younger leaves, the C subgenome was found to be dominant over multiple generations (Bird et al., 2021). The bias to the C subgenome was also found in leaf tissue when the plants were subjected to *Sclerotinia* infection (de Jong & Adams, 2023). Analysis of transcript expression in individual tissues of developing seeds has also highlighted conflicting biases, with A subgenome bias inferred in one experiment (Khan et al., 2022), and later, bias to the C subgenome (Ziegler et al., 2025).

Here, we quantify subgenome expression bias for a set of cultivars of *B. napus* during development and in different tissues. We use the term subgenome dominance to describe various measures of relative expression bias between the A and C subgenomes in a specified cultivar, tissue and developmental context. We investigate subgenome bias using gene expression in apex and leaf tissues across development in three *B. napus* cultivars, with each cultivar representing a distinct crop type. If selective breeding tailored towards climatic conditions results in changes in regulation, different cultivars may undergo differing degrees of adjustment in each subgenome. For instance, a rapid‐cycling cultivar may draw more on the machinery from the A subgenome (Calderwood, Hepworth, et al., 2021). Overall, we find that total gene expression (sum of TPM values) has a larger contribution from the C subgenome. However, in terms of mean expression per gene, the A subgenome is dominant. We find that subgenome bias varies between genotypes, tissues and across development. When we consider only homoeologue gene pairs, we find an expression bias towards the C subgenome across tissues, development and genotypes. On the example of two genes, *FLOWERING LOCUS C* (*FLC*) and *SUPPRESSOR OF OVEREXPRESSION OF CONSTANS 1* (*SOC1*), we discuss the dynamics of homoeologue gene pairs in detail, demonstrating the highly transient nature of expression bias in space and time. Following previous investigations (Bird et al., 2021; de Jong & Adams, 2023; Glombik et al., 2025; Ramírez‐González et al., 2018), we examined the distributions of relative expression levels for the homoeologue gene pairs. We find that the choice of threshold used to define a homoeologue gene pair as biased can influence the results, leading to inconsistent inferences with significant bias switching between subgenomes. The spatio‐temporal variation in expression and the dependence on metrics and thresholds highlight the challenges inherent in quantifying subgenome expression bias.

## RESULTS

### RNA‐seq data from leaf and apex tissue of three *B. napus* cultivars across development provides a comprehensive dataset for studying subgenome bias

To investigate subgenome bias in *B. napus*, we exploited an available RNA‐seq time‐course dataset (Woolfenden et al., 2026) from apex and leaf tissue in three cultivars (Figure 1). Both apex and leaf samples were collected across the vegetative to reproductive transition, starting from young plants (Day 14 after sowing) to the time‐point where first buds became visible (BBCH51). The cultivars in this dataset include a spring type (Stellar DH), a semi‐winter (Zhongshuang 11) and a biennial kale (Ragged Jack). The semi‐winter and biennial kale cultivars both require a period of cold (of different lengths) to induce vernalisation. The mean number of raw reads across all genotypes and tissues was 28.9 M, with minimum and maximum numbers, 24.2 M and 38.5 M, respectively. The reads were aligned to the Darmor‐bzh version 10 genome reference (Rousseau‐Gueutin et al., 2020) with only the uniquely mapped reads used for gene expression quantification. The mean alignment rate for the uniquely mapped reads was 82.5%. The documentation of the dataset (Woolfenden et al., 2026) provides full details regarding the growth conditions, sample collection, sequencing and gene expression quantification.

**Figure 1:**
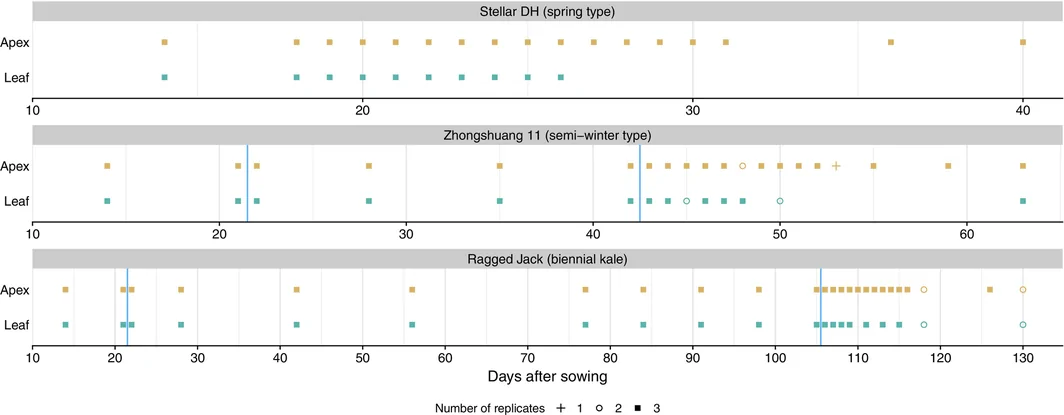
Sample time‐points for RNA‐seq during development for three *Brassica napus* cultivars. Apex (yellow) and leaf (green) tissues were sampled at the indicated time‐points in the spring‐type Stellar DH (top panel), in the semi‐winter type Zhongshuang 11 (ZS) (middle panel), and in the biennial kale, Ragged Jack (RJ) (bottom panel). The number of replicates, 1, 2 or 3, is shown by the shape of the point. The start and end of the period of cold to which ZS and RJ were subjected to induce vernalisation are depicted by the blue vertical lines.

### The C subgenome has a larger contribution to relative transcript abundance than the A subgenome

We first compared the total expression from the A and C subgenomes. For each subgenome, we calculated the total expression as the sum of individual TPM (transcripts per million) values from genes that were annotated as belonging to that subgenome in all time‐series (Figure S1). We computed two metrics to investigate expression bias: the log_2_ ratio of expression from subgenome A over that from subgenome C, log_2_ (A/C), and the relative expression associated with subgenome A, Rel. A = A/(A + C), where A and C here refer to the sum of the TPMs for the respective subgenomes. Both measures are in common use for comparing gene expression. We computed Rel. A for each sample (where sample uniquely identifies the genotype/tissue/time‐point/replicate combination) in all time‐series and computed a Gaussian Process fit (see Materials and Methods), shown in Figure 2. Consistent with Ziegler et al. (2025), we see that the total expression from the C subgenome exceeds that of the A subgenome (Rel. A < 0.5). Figure 2 shows the point‐wise 95% credible intervals of the posterior distributions. As can be seen, the posterior mean is not flat (there is no horizontal line that can be drawn through the credible intervals for all time‐points for any tissue or genotype), demonstrating the dynamic nature of subgenome expression bias. However, the difference in expression between subgenomes is not large: summarising the distributions over time, we find the relative expression values are all slightly below 0.5, with their corresponding log2 fold changes all less than 0.2 (in absolute terms), with the percentage by which C exceeds A under 10% (Table 1).

**Figure 2:**
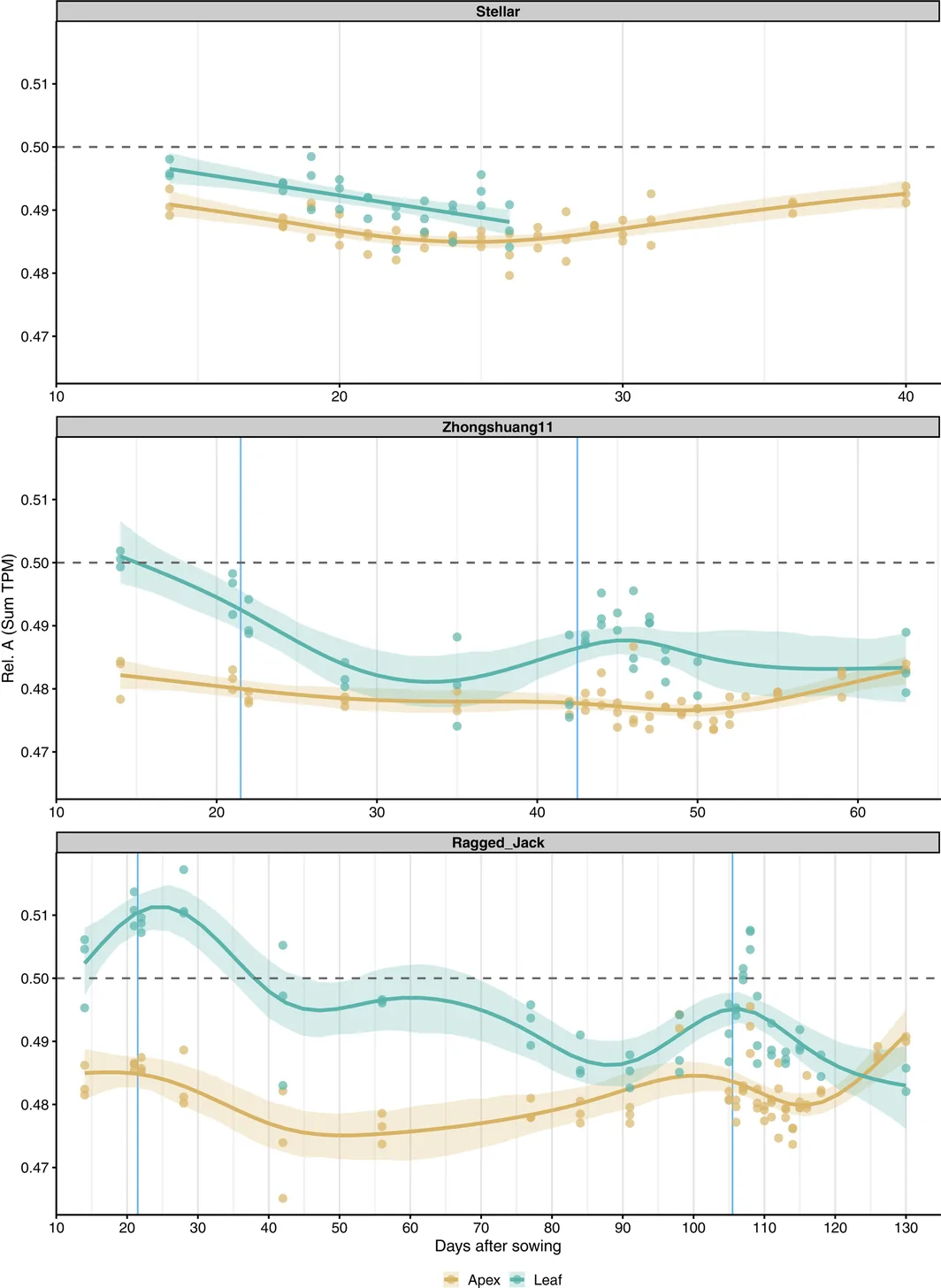
Total gene expression is biased to the C subgenome. These plots show the relative expression, Rel. A = A/(A + C) with A and C referring to the sum of the TPMs for the respective subgenomes, for each subgenome across development, in both tissues and in the indicated cultivars, Stellar, Zhongshuang 11 and Ragged Jack. The apex and leaf data are shown in yellow and green, respectively. The start and end of the period of cold treatment are depicted by the blue vertical lines. Each point represents a sample. The solid line and its surrounding shaded region indicate the point‐wise mean and credible interval from the Gaussian Process fit (see Materials and Methods).

**Table 1:** Summary metrics across the time‐series of subgenome bias based on total expression per subgenome

Cultivar	Tissue	Rel. A	log2FC (A vs C)	Percentage (A vs C)
Stellar	Apex	0.488 (0.487 to 0.489)	−0.068 (−0.072 to −0.065)	4.9% (4.6–5.1%) C>A
Stellar	Leaf	0.492 (0.491 to 0.493)	−0.044 (−0.051 to −0.038)	3.1% (2.6–3.6%) C>A
Zhongshuang 11	Apex	0.479 (0.478 to 0.480)	−0.122 (−0.125 to −0.117)	8.8% (8.5–9.1%) C>A
Zhongshuang 11	Leaf	0.487 (0.485 to 0.488)	−0.077 (−0.088 to −0.068)	5.5% (4.8–6.3%) C>A
Ragged Jack	Apex	0.481 (0.479 to 0.482)	−0.112 (−0.120 to −0.104)	8.1% (7.5–8.6%) C>A
Ragged Jack	Leaf	0.495 (0.494 to 0.497)	−0.029 (−0.037 to −0.020)	2.0% (1.4–2.6%) C>A

*Note*: Rel. A (third column) denotes the relative expression of A, A/(A + C), log2FC is the base 2 logarithm of the fold change (fourth column) and the last column gives the percentage difference in expression between the subgenomes.

We next asked whether the expression bias differed between leaf and apex. We compared the posterior distributions of Rel. A for leaf and apex in each cultivar. In all cases, the 95% credible intervals for the tissue mean (see Materials and Methods) do not overlap (Table 1, third column). Overall, Rel. A was lower, that is, subgenome C is more dominant, in apex (Figure 2, yellow curves) than in the leaf (Figure 2, green curves). However, these results also vary with developmental time, with several instances of overlapping point‐wise credible intervals for which the tissue differences lose statistical significance (approx. day 28 to 38 after sowing and after day 54 for Zhongshuang 11, and Days 85 to 95 and 125 to 130 for Ragged Jack).

Having characterised subgenome bias in terms of total expression between time‐points and between tissues, we evaluated whether these differences manifest themselves in differences between cultivars. Summarising the distributions over time and tissue, we find that the resulting distributions for Rel. A exhibit non‐overlapping 95% credible intervals between cultivars (with the exception of the apex of Zhongshuang 11 and Ragged Jack), suggesting a genotype effect. The C subgenome was dominant in all cultivars (Table 1, third column), but not at all time‐points (Figure 2). Zhongshuang 11 showed the highest bias for subgenome C, whereas Ragged Jack exhibited the largest variation.

In summary, the contribution from the C subgenome to total transcript abundance was overall higher than from the A subgenome of *B. napus*. The subgenome bias showed small but statistically detectable differences over development and between tissues and cultivars. For instance, the subgenome bias in the leaf tissue of the Ragged Jack cultivar switched from A‐biased to C‐biased during development.

### Mean expression per gene is higher for the A subgenome than the C subgenome

The observation that the total TPM contribution from the C subgenome is higher than from the A subgenome is not unexpected given the numbers of annotated genes (59 692 on the C subgenome and 47 193 on the A subgenome, Darmor‐bzh, version 10). To compute the average expression per expressed gene requires setting a threshold for when a gene is expressed; here we used TPM >1. We divided the resulting total TPM values for each subgenome by the number of expressed genes (Figure S2).

Figure 3 shows a Gaussian Process fit of the relative mean expression per gene for leaf and apex in the three cultivars. Normalising by the number of expressed genes reverses the aggregate subgenome bias and introduces subtle modifications to the shape of the mean as the number of expressed genes varies across development. All the observations of the previous section (variability with time, tissue and genotype) hold but now with the opposite inference that the overall bias is towards the A subgenome. The summary results are provided in Table 2.

**Figure 3:**
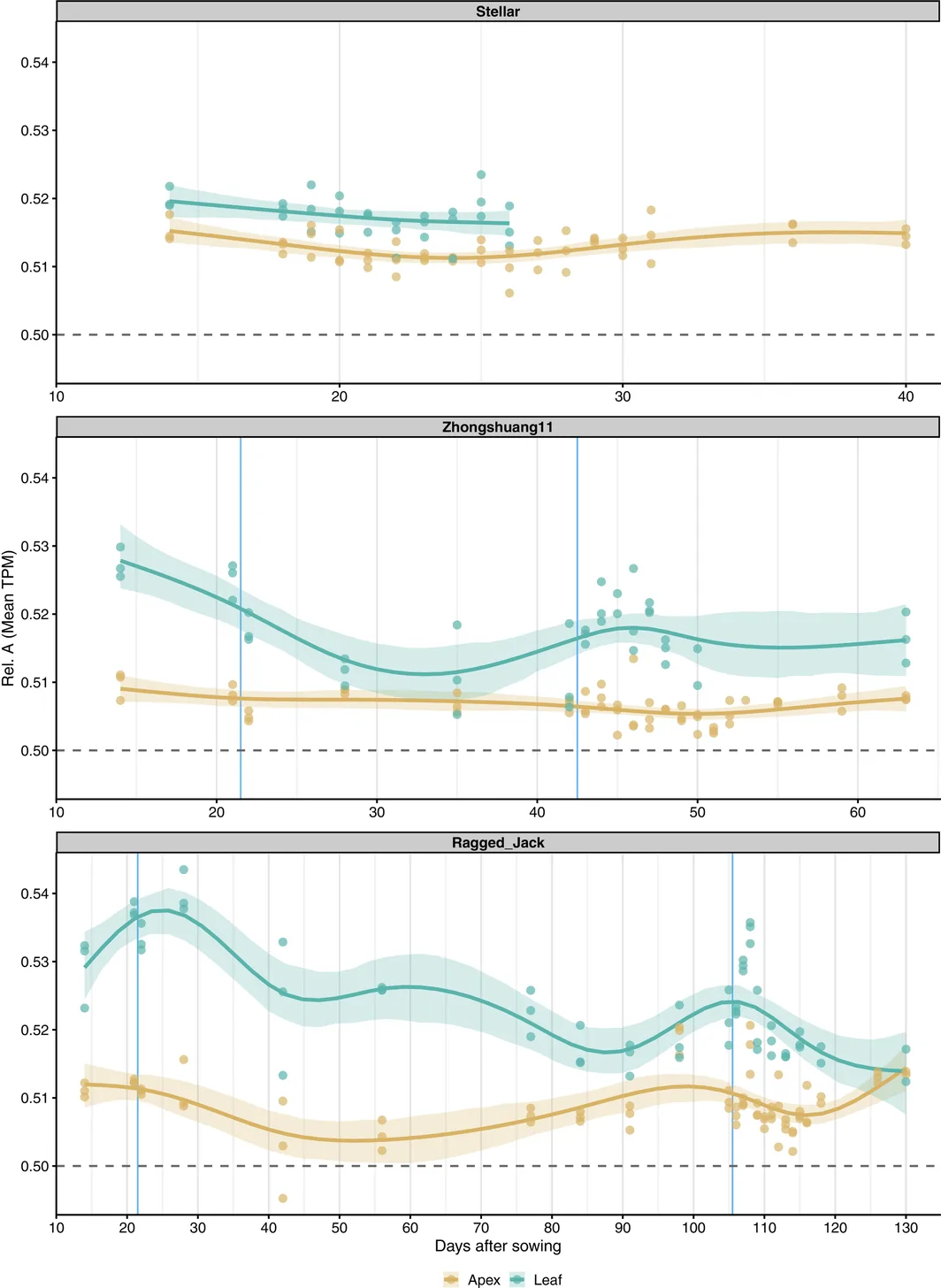
The mean expression per gene is higher for the A subgenome. These plots show the relative expression, Rel. A = A/(A + C) with A and C referring to the mean of the TPMs for the expressed genes in the respective subgenomes, for each subgenome across development, in both tissues and in the indicated cultivars, Stellar, Zhongshuang 11 and Ragged Jack. The apex and leaf data are shown in yellow and green, respectively. The start and end of the period of cold treatment are depicted by the blue vertical lines. Each point represents a sample. The solid line and its surrounding shaded region indicate the point‐wise mean and credible interval from the Gaussian Process fit (see Materials and Methods).

**Table 2:** Summary metrics of subgenome bias based on mean TPM per subgenome

Cultivar	Tissue	Rel. A	log2FC (A vs C)	Percentage (A vs C)
Stellar	Apex	0.513 (0.513 to 0.514)	0.077 (0.073 to 0.081)	5.5% (5.2– 5.8%) A>C
Stellar	Leaf	0.518 (0.517 to 0.519)	0.102 (0.096 to 0.108)	7.3% (6.9–7.7%) A>C
Zhongshuang 11	Apex	0.507 (0.506 to 0.508)	0.040 (0.037 to 0.044)	2.8% (2.6–3.1%) A>C
Zhongshuang 11	Leaf	0.517 (0.515 to 0.518)	0.096 (0.086 to 0.105)	6.9% (6.1–7.5%) A>C
Ragged Jack	Apex	0.508 (0.507 to 0.509)	0.047 (0.041 to 0.053)	3.3% (2.9–3.8%) A>C
Ragged Jack	Leaf	0.524 (0.523 to 0.525)	0.139 (0.131 to 0.147)	10.1% (9.5–10.7%) A>C

*Note*: Rel. A (third column) denotes the relative expression of A, A/(A + C), log2FC is the base 2 logarithm of the fold change (fourth column) and the last column gives the percentage difference in expression between the subgenomes.

In summary, the modest aggregate bias towards the C subgenome for total TPM is reversed to become a modest bias towards the A subgenome when the mean TPM per gene is used to compute the relative expression. We find statistical differences between tissues and between cultivars, but these are subtle and vary across development, thus making inferences conditional on the time of sampling.

### Expression is higher from the C subgenome than the A subgenome in homoeologue gene pairs

Following our observation that the contribution to total expression is higher from the C subgenome yet mean expression per gene is higher for the A subgenome, we sought to directly compare genes common to both subgenomes using homoeologue gene pairs (HGPs).

Figure 4 shows examples of one HGP of *FLOWERING LOCUS C* (*FLC*) and one HGP of *SUPPRESSOR OF OVEREXPRESSION OF CONSTANS 1* (*SOC1*). There are several important observations to make. First, it is noteworthy how transient any specific bias is, with the HGPs exhibiting variations across development that are outside the 95% credible intervals of other developmental stages. Unlike the subgenome‐level aggregate metrics presented in earlier sections for which the bias was subtle, expression bias for HGPs can vary substantially in magnitude and direction. We see several instances (Figure 4a, bottom panel, Figure 4b, middle panel) where the expression of an HGP for *FLC* or *SOC1* changes from strongly biased towards subgenome A to strongly biased towards subgenome C, or *vice versa*. If the sampling had taken place at a time during this transition, the data would have suggested that no significant bias was present at all. Second, we observe a tissue dependence for these HGPs. Thus, the tissue that is being sampled and the time of sampling can drastically change the inferred homoeologue expression bias. Third, there is a pronounced difference in the HGP expression dynamics between cultivars. Given that *FLC* and *SOC1* are sensitive to cold (Maple et al., 2026; Sidhu et al., 2026) and that the cultivars have different vernalisation requirements, these observations may not be surprising. We refer to recent publications on the biological interpretation of the divergent *FLC* and *SOC1* homoeologue dynamics (Calderwood, Lloyd, et al., 2021; Sidhu et al., 2026). These dynamic features are also evident in the expression bias time‐courses for all three HGPs of *FLC* (Figure S3) and all three of *SOC1* (Figure S4).

**Figure 4:**
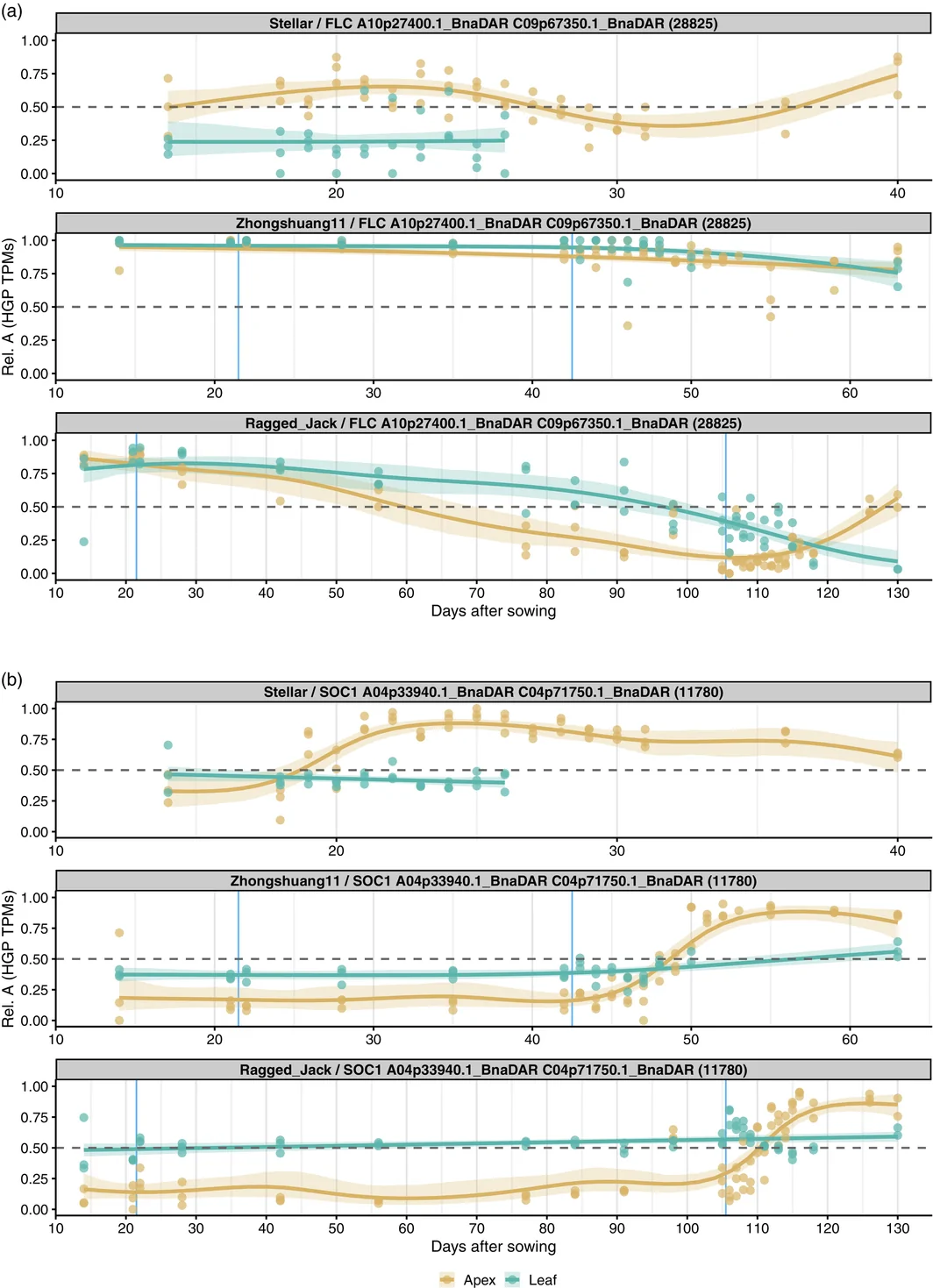
Expression bias is dynamic in homoeologue gene pairs. The relative expression associated with two HGPs, where Rel. A = A/(A + C) is computed from the TPM values of the genes in each HGP in each sample, and in the respective subgenomes across development, in both tissues, and in the indicated genotypes, Stellar, Zhongshuang 11 and Ragged Jack. The relative expression values are shown for one HGP of *FLC* (a), and for one HGP of *SOC1* (b). The apex and leaf data are shown in yellow and green, respectively. The start and end of the period of cold treatment are depicted by the blue vertical lines. Each point represents a sample. The solid line and its surrounding shaded region indicate the point‐wise mean and credible interval from the Gaussian Process fit (see Materials and Methods).

Extending this analysis and using TPM_A_ + TPM_C_ >1, we identified between 16 000 and 19 000 expressed HGPs (depending on the genotype, tissue and time) for further analysis. For each HGP, we summarised the posterior distribution for the associated Gaussian Process fit over time for leaf and apex of the three cultivars. The results are presented in Figure 5. In each case, we see that the bulk of the distribution is approximately centred at 0.5 (equal TPM values for each gene in an HGP), but with the median lower than 0.5 in all cases, indicating that the distributions are biased to the C subgenome. Even for those HGPs with no overall subgenome bias, that is, their credible interval includes 0.5, it is worth remembering that this average value masks the variation that occurs over time. Interestingly, we see several occurrences of HGPs close to 0 or 1, that is, ‘extreme’ HGPs where expression essentially occurs from only one subgenome. This may indicate transcriptional silencing, but could also reflect technical and annotation issues. To quantify how many HGPs fall into the category characterised by extreme Rel. A values, we counted the number of HGPs with credible interval of Rel. A below the 5th or above the 95th percentiles in each distribution (Table 3). Our classification of HGP as exhibiting extreme bias is similar to previous investigations (Bird et al., 2021; de Jong & Adams, 2023; Ziegler et al., 2025) that classified HGP bias into three classes (A‐biased, C‐biased or not biased). Interestingly, even though the distributions showed a bias to the C subgenome, several of the extremely biased sets had more HGPs that were biased to A. We found 1033 HGPs with consistent extreme bias between leaf and apex in Stellar (581 HGPs to A/452 HGPs to C), 890 in Zhongshuang 11 (506 A/384 C) and 780 in Ragged Jack (415 A/365 C).

**Figure 5:**
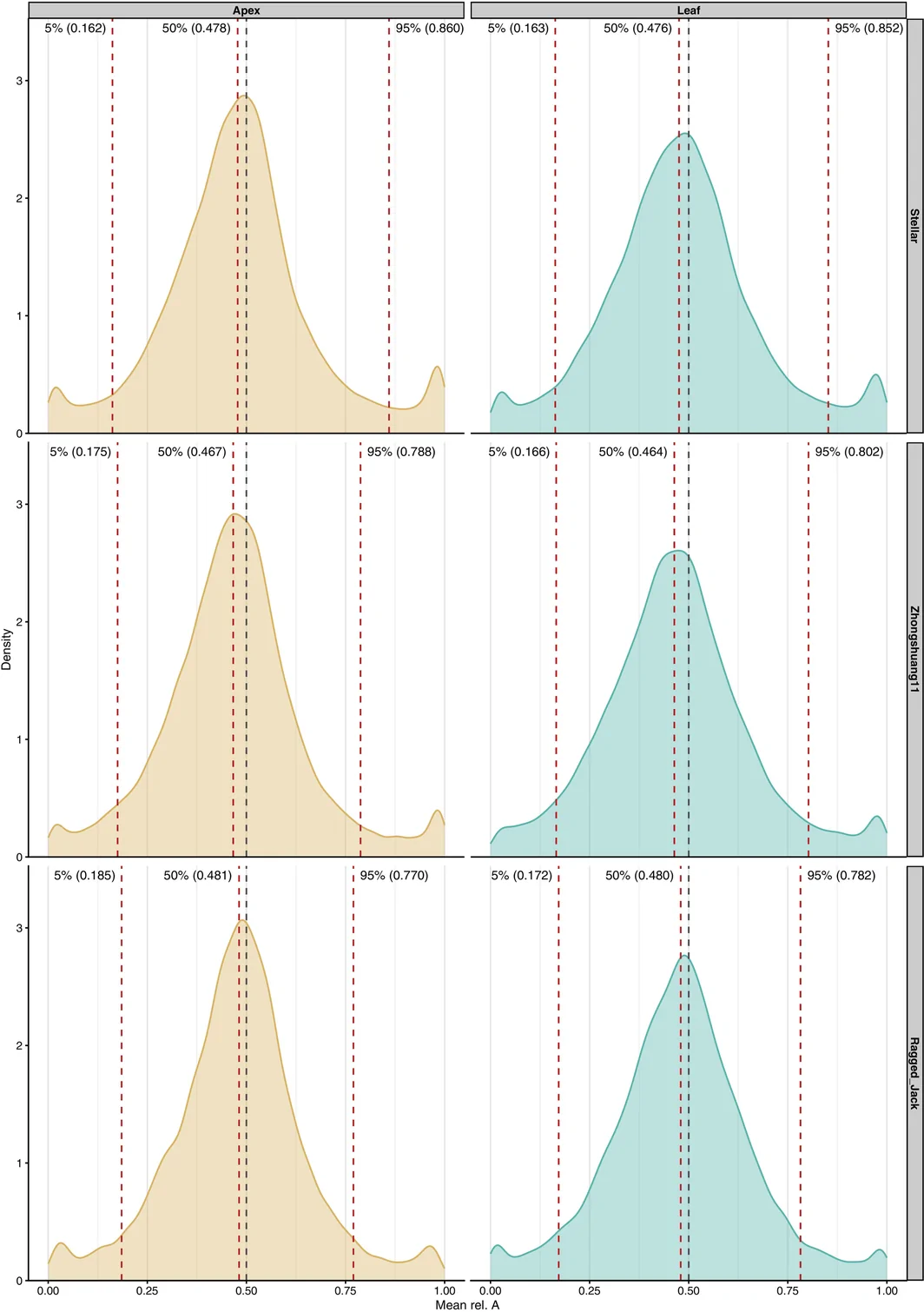
The distributions of the mean relative expression are biased to the C subgenome for the homoeolog gene pairs (HGPs). The mean relative expression across development was calculated from a Gaussian Process fit (see Materials and Methods) for all HGPs that passed an expression filter (TPM_A_ + TPM_C_ > 1) with at least one sample remaining at all time‐points in each time‐series. Each distribution represents the summary over development. The black vertical line indicates where the relative expression of the HGP would be perfectly balanced (Rel. A = 0.5). The red vertical lines indicate the 5th, 50th (median) and 95th percentiles of the distribution. The apex and leaf data are shown in yellow and green, respectively.

**Table 3:** Distribution of extremely biased HGPs

Cultivar	Tissue	Extremely A‐biased	Extremely C‐biased	Not extreme
Stellar	Apex	873	835	16 973
Stellar	Leaf	780	724	15 796
Zhongshuang 11	Apex	849	800	16 965
Zhongshuang 11	Leaf	741	680	15 442
Ragged Jack	Apex	784	788	16 588
Ragged Jack	Leaf	699	680	14 878

*Note*: An HGP is defined as extremely biased if the credible interval of Rel. A is within the 5% and 95% percentiles of the full Rel. A distribution.

To investigate whether specific biological processes, molecular functions or cellular components were enriched in the HGPs that exhibited this extreme expression bias between subgenomes, we performed a GO‐term analysis (see Materials and Methods). In these sets of HGPs, we identified no terms for biological process or molecular function that were significantly enriched (adjusted *P*‐value <0.05) and two significant cellular component terms that were overrepresented, Table S2. There were no significant GO‐term enrichments for Zhongshuang 11, one term for the extremely A‐biased set of Stellar apex (GO term: cell periphery), and one term (GO term: cytosol) for the extremely A‐biased HGPs in both tissues of Ragged Jack. The identified terms do not implicate specific pathways or processes being selectively targeted in terms of subgenome expression bias in HGPs.

Other studies have performed statistics on the numbers of HGPs that were classified as biased based on thresholds for log‐fold changes or Rel. A values. For comparison, we therefore used a range of different thresholds for quantifying HGP expression bias for each sample and investigated the impact of threshold choices (see Materials and Methods). We found that a different fold‐change cut‐off (FCC) could result in samples that were significantly biased to the C subgenome or even reversing the result so that some samples were significantly biased to the A subgenome. We found that this reversal could also happen when we changed either the initial expression filter or the metric that was used to quantify subgenome expression bias. The analysis of numbers of biased HGPs highlights the possibility of different HGP bias classification methods producing different inferences. See Figures S5 and S6, and Table S3 for further detail. These observations, coupled with, in some cases, the highly transient nature of expression bias in HGPs, highlight the importance of considering the full distribution over time (as done above, Figure 5) to obtain a quantification that is less susceptible to temporal fluctuations.

In the previous section, we found that the mean expression per gene is higher for the A subgenome, yet here we observe a bias towards the C subgenome for HGPs. To investigate this seemingly contradictory finding, we repeated the analysis for the mean expression per gene but now distinguishing between genes that are and are not part of an HGP. The results are shown in Figure S8, revealing a further complication to subgenome bias analyses: genes that did not fulfil the criteria for being classified as belonging to an HGP show a strong bias to the A subgenome, whereas genes that are in an HGP exhibit a bias to the C subgenome, that is within the same data there is statistical support for bias towards both the A and the C subgenome.

In summary, on the examples of *FLC* and *SOC1*, we found that the expression dynamics within an HGP can be dramatic, varying from C‐biased to unbiased to A‐biased across development. We identified several hundred HGPs that exhibit extreme bias (i.e., one gene in the HGP expressed and the other either not or lowly expressed); although these HGPs differed between cultivars, we failed to identify statistically significant biological patterns in these sets. In all distributions of relative expression in HGPs, we identified a subtle bias towards the C subgenome, Figure 5. The discrepancy between A subgenome bias for the mean expression per gene and the C subgenome bias for HGPs can be explained by the contributions of different subsets (genes in a HGP and genes not in a HGP) from each subgenome, Figure S8.

## DISCUSSION

The aftermath of two progenitor genomes clashing together in a hybridisation event has been shown to cause structural rearrangements of the genome, silencing of transposons and epigenetic modifications (Session & Rokhsar, 2023), leading to heritable regulatory adjustments and changes in expression. A model system for studying polyploidisation is the allotetraploid *B. napus* which is formed from its progenitors *B. rapa* (subgenome A) and *B. oleracea* (subgenome C). In such systems, an interesting question is whether the phenotype of the polyploid can be explained in terms of the characteristics of its parts, that is, the subgenomes, and whether one ancestral genome contributes more than the other. In *B. napus*, several asymmetries between subgenomes are apparent: the A subgenome has more chromosomes (10) than the C subgenome (9); the C subgenome has more genes (approx. 59 000, the number depending on the annotation software and the cultivar) than the A subgenome (approx. 47 000); the C subgenome is significantly larger (525 Mb) and has a higher percentage of transposable elements (53%) compared to the A subgenome (315 Mb and 38% transposable elements). Whilst these measures, along with the numbers of retained genes relative to the parental genomes, may vary between cultivars, they are static within the lifetime of a plant and common across tissues.

In contrast, several measures of subgenome dominance are focussed on expression. As gene expression is dynamic, varying with genotype, tissue and developmental stage, we hypothesised that expression‐based metrics of subgenome bias may be context‐ and time‐dependent. Subgenome dominance in *B. napus* has been analysed in depth, yet with seemingly conflicting results between studies, raising the question of whether the different metrics and context‐dependent, dynamic nature of expression may explain these discrepancies.

Here, we evaluated different metrics for quantifying the extent of subgenome A or subgenome C dominance in *B. napus* over developmental time in the leaf and apex of three different cultivars that represent distinct crop types. We note that for Ragged Jack and Zhongshuang 11 RNA‐seq was performed using the Illumina HiSeq X platform, whereas for Stellar on the Illumina NovaSeq 6000 platform. Also, all three cultivars were mapped to the Darmor‐bzh version 10 genome reference (Rousseau‐Gueutin et al., 2020) and that cultivar‐specific structural variation or sequence divergence may have introduced differing alignment rates between the cultivars and their subgenomes. However, the QC metrics were high (Woolfenden et al., 2026) and by using stringent filters we reduced the risk of artefacts. We investigated aggregate metrics, such as total expression and mean gene expression, as well as gene‐based metrics, such as homoeologous expression bias and the number of biased homoeologue gene pairs.

We found that the total gene expression per subgenome was slightly larger (between 2.0 and 8.8%) from the C subgenome than from the A subgenome, consistent with the observations of Ziegler et al. (2025) in individual tissues of the developing *B. napus* seed. This bias in total gene expression between subgenomes A and C was different (non‐overlapping 95% credible intervals of the posterior distribution) between tissues and between cultivars. Normalising the total gene expression per subgenome by the number of expressed genes for each subgenome, however, reveals a higher mean gene expression for genes from the A subgenome, between 2.8 and 10.1% (on average) higher than C. The lower mean expression per gene from the C subgenome may reflect the greater level of transposon density and epigenetic silencing affecting neighbouring genes. The mean expression per gene showed credible differences between tissues within each cultivar, and between cultivars for each tissue. The metrics of total expression and mean expression per gene thus result in opposing biases towards subgenomes. Thus, at the subgenome level, we have various metrics with seemingly contrasting biases, but they are all measuring subtly different things.

To obtain a more detailed view, we investigated homoeologue gene pairs (HGPs). As parallel genes being retained on the progenitor‐derived subgenomes, HGPs allow direct comparison between A and C subgenome expression. The examples of *SOC1* and *FLC*, Figure 4, Figures S3 and S4, show that homoeologue expression bias can vary dramatically over time, transiently going from one extreme to another, for example, from A‐copy dominance to C‐copy dominance or *vice versa*. These observations strikingly demonstrate the fluid nature of subgenome bias. Had the sampling taken place at a single time‐point, the inferred direction and magnitude of expression bias would have depended on which day the tissue happened to be collected. These insights are perhaps not surprising as gene expression is highly dynamic and tissue‐specific, in line with our hypothesis that measures of subgenome bias based on expression can vary within a single plant. Indeed, as has been previously reported, the dominant subgenome may in fact be more lowly expressed at specific developmental time‐points (Alger & Edger, 2020), thus highlighting the importance of examining trends between tissues and across plant development.

The expression over time between genes in homoeologue gene pairs (HGPs) revealed a bias to the C subgenome. Thus, whilst the mean expression per gene is higher for genes from the A subgenome, the HGPs exhibit a higher expression for genes from the C subgenome. These seemingly conflicting expression biases suggest that genes that are not part of a HGP must have an even greater expression bias to the A subgenome to produce the average that we observe (Figure S8). The observation that 86.7% of HGPs in *B. napus* have similar expression to corresponding genes in their diploid parents (Li et al., 2020) suggests that the observed bias could be linked to the life histories of the progenitors and their evolution prior to the hybridisation event, rather than the ‘transcriptome shock’ experienced upon polyploidisation or subsequent adaptation. Resynthesised cultivars that ‘replay the tape’ (e.g. Bird et al., 2021) are an excellent route for studying the latter, with the inclusion of further organs, tissues and/or time‐points augmenting the spatio‐temporal picture of subgenome bias. Combined with cultivar‐specific genome references, such data could underpin the investigation of regulatory elements that are causally implicated in gene expression (Rockenbach et al., 2025).

In summary, depending on which metric was used, we found evidence for subgenome expression bias towards both the A and the C subgenomes of *B. napus*. All metrics revealed differences between apex and leaf tissue, and between cultivars for a particular tissue. However, the differences in all aggregate bias metrics were modest, context‐dependent and dynamic. These results highlight the importance of examining subgenome expression not only across development and within tissues (Alger & Edger, 2020) but also between cultivars. The tissue‐ and genotype‐specific dependencies coupled with expression dynamics introduce challenges for comparing results on subgenome bias between studies. For instance, previous work has demonstrated a bias to the A subgenome (Li et al., 2020; Wu et al., 2018) whereas other reports identified a bias to the C subgenome (Bird et al., 2021; de Jong & Adams, 2023). Comparisons are further complicated by the use of different expression filters and thresholds. To classify an HGP as biased requires the introduction of a threshold for the difference in expression. We found that the number of biased HGPs is sensitive to the classification method and the chosen thresholds, leading us to favour robust statistical quantification over classification. We used relative expression (or expression probability), A/(A + C), to quantify differential expression between subgenomes. This metric is less sensitive to numerical instabilities and distortions introduced by log‐fold change or pseudo log measures. Here, we calculated the relative expression using TPM values but point out that RNA‐seq counts, normalised by gene‐length, offer several advantages in terms of Bayesian analyses (depending on the number of subgenomes, a Beta or Dirichlet distribution is a convenient choice for capturing the distribution of the expression probability, leading to Beta‐Binomial or Dirichlet‐Multinomial posterior distributions, respectively). Hierarchical (multi‐level) Bayesian approaches provide an effective means of sharing information between samples and capturing dependencies in the data, yet come at a high computational cost. We therefore used Gaussian Processes (coupled with appropriate transformations) for analysing time‐series data. A detailed discussion of these different approaches will be presented elsewhere.

The observed dynamics of *FLC* and *SOC1* HGPs offer an interesting direction for future work to mechanistically unravel the bias switching, that is, whether this is linked to specific *cis*‐regulatory elements, epigenetics, chromatin changes, or trans‐regulation associated with vernalisation and the floral transition. Understanding when and how individual HGPs change their expression and switch dominance during development, and the phenotypic implications may provide key insights into adaptation and suggest targeted routes for exploiting these findings for crop breeding programmes.

## MATERIALS AND METHODS

### Plant materials and growth conditions

The three *B. napus* cultivars Stellar DH (a spring oilseed rape, OSR), Zhongshuang 11 (a semi‐winter OSR) and Ragged Jack (a heritage biennial kale) were grown in P24 tray modules in cereal mix (40% medium grade peat, 40% sterilised soil, 20% horticultural grit, 1.3 kg m^−3^ PG mix 14‐16‐18 + Te Base Fertiliser, 1 kg m^−3^ Osmocote Mini 16‐8‐11 2 mg + Te 0.02% B, wetting agent, 3 kg m^−3^ maglime, 300 g m^−3^ Exemptor). Plants were grown in a Conviron MTPS 144 controlled environmental room with Valoya NS1 LED lighting (250 μmol m^−2^ sec^−1^) 18°C/15°C day/night temperatures, 70% relative humidity with a 16‐hour day. At 21 days, the plants from the semi‐winter and biennial kale cultivars were transferred to vernalising conditions with the temperature set to 5°C, an 8‐hour photoperiod and fluorescent lighting (60 μmol m^−2^ sec^−1^). After 3 weeks (Zhongshuang 11) or 12 weeks (Ragged Jack) of cold treatment, plants were returned to the controlled environment rooms until maturity. Further details are available in the time‐course dataset descriptor (Woolfenden et al., 2026).

### Sample preparation

Leaves (first true leaf) and apex tissues from 14 days after sowing and onwards were collected from each *Brassica* genotype until BBCH51 when floral buds became visible at the plant apex. Three biological replicates, comprising pooled tissue from three individuals, were sampled for each time‐point and tissue type, except where stated otherwise. Tissue was immediately frozen in liquid nitrogen and stored at −80°C prior to RNA extraction.

### RNA extraction and sequencing details

Tissue was ground to a fine powder in liquid nitrogen in an autoclaved mortar and pestle (leaf) or Eppendorf and micro pestle (apex). RNA extraction and DNase treatment were performed following the method provided with the EZNA® Plant RNA Kit (Omega Biotek Inc., http://omegabiotek.com/store). RNA integrity number (RIN) was determined using an Agilent 2100 Bioanalyzer (Agilent Technologies, Inc.). Total RNA samples were processed by Novogene (Beijing, China) to produce NEB ultra directional stranded libraries for 150 bp paired‐end sequencing. Ragged Jack and Zhongshuang 11 sequencing was performed using the Illumina HiSeq X platform and Stellar was sequenced on the Illumina NovaSeq 6000 platform.

### Gene expression data

The sequenced data were first trimmed and cleaned with Trimmomatic (v0.39) (Bolger et al., 2014) and then aligned to the Darmor‐bzh version 10 genome reference (Rousseau‐Gueutin et al., 2020) using HISAT2 (v2.1.0) (Kim et al., 2019) with additional options ‘‐‐pen‐noncansplice 20 ‐‐mp 1,0’ (Baruzzo et al., 2017). Uniquely mapped reads were extracted with samtools (v1.17) (Danecek et al., 2021) using the ‘‐‐tag NH:1’ tag and gene expression quantification was obtained using StringTie (v2.1.1) (Pertea et al., 2016). The mean number of raw reads across all genotypes and tissues was 28.9 M reads, with minimum and maximum numbers, 24.2 M and 38.5 M, respectively. The mean alignment rate for the uniquely mapped reads was 82.5%. The documentation of the dataset (Woolfenden et al., 2026) provides additional details, including the read alignment script and gene expression quantification.

### Statistical methods

We used R v4.5.3 (The R Foundation, 2023) for all analyses. We used the ‘gam’ function of the ‘mgcv’ R package (v1.9–4) (Wood, 2017) to fit a generalised additive model (GAM) to a time‐series with Gaussian Process‐based smoothing (bs = ‘gp’). When fitting Rel. A time‐series we used the beta regression (‘betar’) family with a ‘logit’ link because Rel. A is a proportion (between 0 and 1) and so can be modelled using a beta distribution. There are numerical issues if there are values at 0 or 1 so we applied the Smithson‐Verkuilen transformation (Smithson & Verkuilen, 2006) if any of the input values were close (within machine precision, sqrt(.Machine$double.eps)) to these values. We then performed empirical Bayes' sampling from the fit (using the ‘rmvn’ function in the ‘mgcv’ package) to compute the posterior distributions across the time‐course with 100 equally‐spaced time‐points and 250 posterior simulations. From these we computed the point‐wise means and 95% credible intervals (CrI), which are shown in the plots. We then summarised the point‐wise data to obtain the mean and CrI for the time‐series, with these summary values shown in Tables 1 and 2. For each posterior simulation, we back‐transformed the logit‐scale predictor to the Rel. A scale before computing the posterior samples. When fitting the raw time‐series data, for example, the sum of the TPMs per subgenome (Figure S1), we used ‘gaussian’ (with ‘identity’ link) as the family in ‘gam’. The empirical Bayes' sampling and the computation of the point‐wise means and CrI remained the same, but without the need for logit transformations. We used reduced maximum likelihood (REML) for the smoothing parameter estimation throughout. We used ggplot2 v4.0.2 (Wickham, 2016) to plot all figures.

### Homoeologue identification

To identify homoeologue gene pairs (HGPs) in the Darmor‐bzh v10 genome reference (Rousseau‐Gueutin et al., 2020) we first split the genome reference by subgenome and ran the mmseqs2 (Steinegger & Söding, 2017) reciprocal best hit pipeline (‘easy‐rbh’) between the A and C subgenomes for both the nucleotide and protein sequences with parameters ‘‐s 7.5 ‐c 0.6’. This resulted in 31 214 and 32 868 potential HGPs for the nucleotide and protein sequences, respectively, and a combined total of 34 713. To account for homoeologous exchange between the subgenomes we used GENESPACE v1.3.1 (Lovell et al., 2022) to identify syntenic hits and used the results to filter the list of potential HGPs to obtain the 29 336 HGPs used in the analysis. The chromosome‐by‐chromosome HGP counts are shown in Figure S7 and listed in Table S1.

### Homoeologue gene pair (HGP) bias classification

HGP bias classification followed a three‐step process. First, the set of HGPs was obtained by filtering on the expression of the two genes in the pair. Following previous investigations, we used two filtering criteria: both genes in the pair had TPM values larger than one (labelled ‘A > 1 & C > 1’); the sum of both TPM values was larger than two (labelled ‘A + C > 2’). The sum filter is less stringent and admits all HGPs that pass the individual TPM filter, while also including HGPs with lowly expressed genes. The second step assessed the numerical value of the relative expression. We used different methods that have all been used in previous work: the logarithm of the fold change $\left(\log_{2}\left[\mathrm{TP}M_{A}/\mathrm{TP}M_{C}\right]\right)$, labelled ‘log A/C’; a ‘pseudo‐TPM’ log‐fold change $\left(\log_{2}\left(\left[\mathrm{TP}M_{A}+1\right]/\left[\mathrm{TP}M_{C}+1\right]\right)\right)$, ‘pseudo‐log A/C’; a relative expression value in the range zero to one ($\mathrm{TP}M_{A}/\left[\mathrm{TP}M_{A}+\mathrm{TP}M_{C}\right])$, ‘Rel. A’. The log‐fold change approaches have been used in *B. napus* (Bird et al., 2021; de Jong & Adams, 2023; Ziegler et al., 2025) and the ‘Rel. A’ method in wheat (Glombik et al., 2025; Ramírez‐González et al., 2018). Third, the relative expression is compared to a fold‐change cut‐off (FCC) to classify whether the HGP is biased and to which subgenome, that is, A‐biased, C‐biased or not biased. For example, values produced by the ‘log A/C’ method that are larger than $\log_{2}\left(\mathrm{FCC}\right)$ are labelled A‐biased while values lower than $-\log_{2}\left(\mathrm{FCC}\right)$ are labelled C‐biased. We used the values 2, 3 and 4 for the FCC. The FCC can be transformed to a value suitable for the ‘Rel. A’ method by the formula, rFCC = FCC/(FCC + 1), where rFCC is the ‘Rel. A FCC’. For example, an FCC of 3 would have a rFCC of 3/4 so that HGPs with ‘Rel. A’ above this would be labelled as A‐biased, with HGPs that have a rFCC below $1/4\;\left(=1-3/4\right)$ labelled as C‐biased. It should be noted that for the ‘A > 1 & C > 1’ filter the methods ‘log A/C’ and ‘Rel. A’ are interchangeable and so produce the same bias classes. We thus used two different filters for expression, three methods to calculate the relative expression, and three different FCC values to investigate the robustness of each one by comparing their outcomes across a time‐course. For each sample we counted the number of HGPs that were biased to each subgenome and performed a statistical test (Chi‐squared test) to check whether the number of HGPs significantly deviated from parity (see Results, Discussion, Figures S5 and S6).

### Gene ontology (GO) analysis

We performed GO analysis using g:Profiler (Kolberg et al., 2023) (version e114_eg62_p19_27110d83) with the default settings, for example, an adjusted *P*‐value threshold of 0.05. For input, we used the *Arabidopsis thaliana* genes corresponding to the genes of interest. For HGPs we ensured that both genes in the pair mapped to the same Arabidopsis gene. We performed the homology lookup from *B. napus* to Arabidopsis using the homology data downloaded from PLAZA Plants (version 5) (Bel et al., 2021), from where we also downloaded the Darmor‐bzh v10 (Rousseau‐Gueutin et al., 2020) genome reference. We filtered the homology data such that each *B. napus* gene mapped to a single Arabidopsis gene. The background set of *B. napus* genes was obtained by only taking those *B. napus* genes that were expressed (TPM >1) at least once during the relevant time‐course (e.g. Stellar/Apex). We then obtained the Arabidopsis gene for these genes from the homology data. The fold enrichment is not included explicitly in the g:Profiler output but can be calculated by dividing the observed frequency (intersection size/query size) by the expected frequency (GO‐term size/effective domain size) where the terms in parentheses are available in the g:Profiler results.

## AUTHOR CONTRIBUTIONS

RJM and RW conceived the project. HW performed the analysis and drafted the manuscript. All authors contributed to writing the manuscript. The authors read and approved the final manuscript.

## CONFLICT OF INTEREST

The authors declare that they have no competing interests.

## AI STATEMENT

We used Gemini (Pro 3.1 and Flash 3.6) to assist with R scripting. The authors are responsible for all content. No biological interpretations were generated by AI.

## Supporting information


**Figure S1.** Total gene expression is biased to the C subgenome.
**Figure S2.** Mean expression of the genes shows that A subgenome dominates.
**Figure S3.** The relative expression for the homoeologue gene pairs (HGPs) of *FLOWERING LOCUS C* (FLC) is dynamic.
**Figure S4.** The relative expression for the homoeologue gene pairs (HGPs) of SOC1 is dynamic.
**Figure S5.** Comparison of subgenome bias classification methods to determine their impact on detecting bias.
**Figure S6.** Assessing the effect of different methods of homoeologue gene pairs (HGP) bias classification across the apex time‐course in Stellar.
**Figure S7.** Number of homoeologue gene pairs (HGPs) between pairs of chromosomes on the two subgenomes.
**Figure S8.** The mean expression per gene depends on whether the genes are part of a HGP, or not.

**Table S1.** List of homoeologue gene pairs.

**Table S2.** List of significant gene ontology (GO) terms for the extremely biased homoeologue gene pairs (HGPs).

**Table S3.** Counts of biased homoeologue gene pairs (HGPs) for each bias classification approach together with the number of HGPs in common.

## Data Availability

The raw RNA‐seq data (FASTQ files) have been deposited in the European Nucleotide Archive (ENA) under accession numbers PRJEB106353 (Stellar), PRJEB106354 (Zhongshuang 11) and PRJEB106340 (Ragged Jack). Full details of the dataset are given in (Woolfenden et al., 2026), which also provides the expression data for each genotype.

## References

[tpj71145-bib-0001] Alger, E.I. & Edger, P.P. (2020) One subgenome to rule them all: underlying mechanisms of subgenome dominance. Current Opinion in Plant Biology, 54, 108–113. Available from: 10.1016/j.pbi.2020.03.004 32344327

[tpj71145-bib-0002] Baruzzo, G. , Hayer, K.E. , Kim, E.J. , Camillo, B.D. , FitzGerald, G.A. & Grant, G.R. (2017) Simulation‐based comprehensive benchmarking of RNA‐seq aligners. Nature Methods, 14, 135–139. Available from: 10.1038/nmeth.4106 PMC5792058 27941783

[tpj71145-bib-0003] Bel, M.V. , Silvestri, F. , Weitz, E.M. , Kreft, L. , Botzki, A. , Coppens, F. et al. (2021) PLAZA 5.0: extending the scope and power of comparative and functional genomics in plants. Nucleic Acids Research, 50, D1468–D1474. Available from: 10.1093/nar/gkab1024 PMC8728282 34747486

[tpj71145-bib-0004] Bird, K.A. , Niederhuth, C.E. , Ou, S. , Gehan, M. , Pires, J.C. , Xiong, Z. et al. (2021) Replaying the evolutionary tape to investigate subgenome dominance in allopolyploid *Brassica napus* . New Phytologist, 230, 354–371. Available from: 10.1111/nph.17137 PMC7986222 33280122

[tpj71145-bib-0005] Bird, K.A. , VanBuren, R. , Puzey, J.R. & Edger, P.P. (2018) The causes and consequences of subgenome dominance in hybrids and recent polyploids. New Phytologist, 220, 87–93. Available from: 10.1111/nph.15256 29882360

[tpj71145-bib-0006] Bolger, A.M. , Lohse, M. & Usadel, B. (2014) Trimmomatic: a flexible trimmer for Illumina sequence data. Bioinformatics, 30, 2114–2120. Available from: 10.1093/bioinformatics/btu170 PMC4103590 24695404

[tpj71145-bib-0007] Calderwood, A. , Hepworth, J. , Woodhouse, S. et al. (2021) Comparative transcriptomics reveals desynchronisation of gene expression during the floral transition between Arabidopsis and *Brassica rapa* cultivars. Quantitative Plant Biology, 2, e4. Available from: 10.1017/qpb.2021.6 PMC10095958 37077206

[tpj71145-bib-0008] Calderwood, A. , Lloyd, A. , Hepworth, J. et al. (2021) Total *FLC* transcript dynamics from divergent paralogue expression explains flowering diversity in *Brassica napus* . New Phytologist, 229, 3534–3548. Available from: 10.1111/nph.17131 PMC7986421 33289112

[tpj71145-bib-0009] Chalhoub, B. , Denoeud, F. , Liu, S. et al. (2014) Early allopolyploid evolution in the post‐Neolithic *Brassica napus* oilseed genome. Science, 345, 950–953. Available from: 10.1126/science.1253435 25146293

[tpj71145-bib-0010] Conant, G.C. , Birchler, J.A. & Pires, J.C. (2014) Dosage, duplication, and diploidization: clarifying the interplay of multiple models for duplicate gene evolution over time. Current Opinion in Plant Biology, 19, 91–98. Available from: 10.1016/j.pbi.2014.05.008 24907529

[tpj71145-bib-0011] Conant, G.C. & Wolfe, K.H. (2008) Turning a hobby into a job: how duplicated genes find new functions. Nature Reviews Genetics, 9, 938–950. Available from: 10.1038/nrg2482 19015656

[tpj71145-bib-0012] Danecek, P. , Bonfield, J.K. , Liddle, J. et al. (2021) Twelve years of SAMtools and BCFtools. GigaScience, 10, giab008. Available from: 10.1093/gigascience/giab008 PMC7931819 33590861

[tpj71145-bib-0013] de Jong, G.W. & Adams, K.L. (2023) Subgenome‐dominant expression and alternative splicing in response to *Sclerotinia* infection in polyploid *Brassica napus* and progenitors. The Plant Journal, 114, 142–158. Available from: 10.1111/tpj.16127 36710652

[tpj71145-bib-0014] Fang, C. , Jiang, N. , Teresi, S.J. , Platts, A.E. , Agarwal, G. , Niederhuth, C. et al. (2024) Dynamics of accessible chromatin regions and subgenome dominance in octoploid strawberry. Nature Communications, 15, 2491. Available from: 10.1038/s41467-024-46861-0 PMC10954716 38509076

[tpj71145-bib-0015] Glombik, M. , Arunkumar, R. , Burrows, S. , Mogg, S.L. , Wang, X. & Borrill, P. (2025) Rapid reprogramming and stabilization of homoeolog expression bias in hexaploid wheat biparental populations. Genome Biology, 26, 147. Available from: 10.1186/s13059-025-03598-3 PMC12121048 40437599

[tpj71145-bib-0016] Glover, N.M. , Redestig, H. & Dessimoz, C. (2016) Homoeologs: what are they and how do we infer them? Trends in Plant Science, 21, 609–621. Available from: 10.1016/j.tplants.2016.02.005 PMC4920642 27021699

[tpj71145-bib-0017] Grover, C.E. , Gallagher, J.P. , Szadkowski, E.P. , Yoo, M.J. , Flagel, L.E. & Wendel, J.F. (2012) Homoeolog expression bias and expression level dominance in allopolyploids. New Phytologist, 196, 966–971. Available from: 10.1111/j.1469-8137.2012.04365.x 23033870

[tpj71145-bib-0018] Hegarty, M.J. , Barker, G.L. , Wilson, I.D. , Abbott, R.J. , Edwards, K.J. & Hiscock, S.J. (2006) Transcriptome shock after interspecific hybridization in *Senecio* is ameliorated by genome duplication. Current Biology, 16, 1652–1659. Available from: 10.1016/j.cub.2006.06.071 16920628

[tpj71145-bib-0019] Jiao, Y. , Wickett, N.J. , Ayyampalayam, S. et al. (2011) Ancestral polyploidy in seed plants and angiosperms. Nature, 473, 97–100. Available from: 10.1038/nature09916 21478875

[tpj71145-bib-0020] Khan, D. , Ziegler, D.J. , Kalichuk, J.L. , Hoi, V. , Huynh, N. , Hajihassani, A. et al. (2022) Gene expression profiling reveals transcription factor networks and subgenome bias during *Brassica napus* seed development. The Plant Journal, 109, 477–489. Available from: 10.1111/tpj.15587 34786793

[tpj71145-bib-0021] Kim, D. , Paggi, J.M. , Park, C. , Bennett, C. & Salzberg, S.L. (2019) Graph‐based genome alignment and genotyping with HISAT2 and HISAT‐genotype. Nature Biotechnology, 37, 907–915. Available from: 10.1038/s41587-019-0201-4 PMC7605509 31375807

[tpj71145-bib-0022] Kolberg, L. , Raudvere, U. , Kuzmin, I. , Adler, P. , Vilo, J. & Peterson, H. (2023) g:Profiler—interoperable web service for functional enrichment analysis and gene identifier mapping (2023 update). Nucleic Acids Research, 51, W207–W212. Available from: 10.1093/nar/gkad347 PMC10320099 37144459

[tpj71145-bib-0023] Li, M. , Wang, R. , Wu, X. & Wang, J. (2020) Homoeolog expression bias and expression level dominance (ELD) in four tissues of natural allotetraploid *Brassica napus* . BMC Genomics, 21, 330. Available from: 10.1186/s12864-020-6747-1 PMC7191788 32349676

[tpj71145-bib-0024] Lovell, J.T. , Sreedasyam, A. , Schranz, M.E. , Wilson, M. , Carlson, J.W. , Harkess, A. et al. (2022) GENESPACE tracks regions of interest and gene copy number variation across multiple genomes. eLife, 11, e78526. Available from: 10.7554/elife.78526 PMC9462846 36083267

[tpj71145-bib-0025] Maple, R. , Menon, G. , Duncan, S. , Schulten, A. , Sindalovskaya, M. , Yang, H. et al. (2026) Co‐transcriptional splicing changes combine with reduced productive transcription initiation for cold‐induced repression of *FLC* . Nucleic Acids Research, 54, gkaf1462. Available from: 10.1093/nar/gkaf1462 PMC12781886 41505085

[tpj71145-bib-0026] Morris, J.P. , Baslan, T. , Soltis, D.E. , Soltis, P.S. & Fox, D.T. (2024) Integrating the study of polyploidy across organisms, tissues, and disease. Annual Review of Genetics, 58, 297–318. Available from: 10.1146/annurev-genet-111523-102124 PMC11590481 39227132

[tpj71145-bib-0027] Pertea, M. , Kim, D. , Pertea, G.M. , Leek, J.T. & Salzberg, S.L. (2016) Transcript‐level expression analysis of RNA‐seq experiments with HISAT, StringTie and ballgown. Nature Protocols, 11, 1650–1667. Available from: 10.1038/nprot.2016.095 PMC5032908 27560171

[tpj71145-bib-0028] Ramírez‐González, R.H. , Borrill, P. , Lang, D. , Harrington, S.A. , Brinton, J. , Venturini, L. et al. (2018) The transcriptional landscape of polyploid wheat. Science, 361, eaar6089. Available from: 10.1126/science.aar6089 30115782

[tpj71145-bib-0029] Rapp, R.A. , Udall, J.A. & Wendel, J.F. (2009) Genomic expression dominance in allopolyploids. BMC Biology, 7, 18. Available from: 10.1186/1741-7007-7-18 PMC2684529 19409075

[tpj71145-bib-0030] Rockenbach, K.C. , Zanini, S.F. , Tidy, A.C. , Morris, R.J. , Wells, R. & Golicz, A.A. (2025) A deep learning model captures position‐specific effects of plant regulatory sequences and suggests genes under complex regulation. *bioRxiv: The Preprint Server for Biology*, 2025.08.30.673246. Available at: 10.1101/2025.08.30.673246 PMC13436691 42413033

[tpj71145-bib-0031] Rousseau‐Gueutin, M. , Belser, C. , Silva, C.D. , Richard, G. , Istace, B. , Cruaud, C. et al. (2020) Long‐read assembly of the *Brassica napus* reference genome Darmor‐bzh. GigaScience, 9, giaa137. Available from: 10.1093/gigascience/giaa137 PMC7736779 33319912

[tpj71145-bib-0032] Sankoff, D. & Zheng, C. (2012) Fractionation, rearrangement and subgenome dominance. Bioinformatics, 28, i402–i408. Available from: 10.1093/bioinformatics/bts392 PMC3436819 22962459

[tpj71145-bib-0033] Session, A.M. & Rokhsar, D.S. (2023) Transposon signatures of allopolyploid genome evolution. Nature Communications, 14, 3180. Available from: 10.1038/s41467-023-38560-z PMC10235133 37263993

[tpj71145-bib-0034] Sidhu, G.S. , Burrows, S. , Woolfenden, H. , Wells, R. & Morris, R.J. (2026) Network analysis of flowering time genes suggests regulatory changes among *SOC1* orthologues in response to cold in *Brassica napus* . Journal of Experimental Botany, erag420. Available from: 10.1093/jxb/erag420 42663034

[tpj71145-bib-0101] Smithson, M. & Verkuilen, J. (2006) A better lemon squeezer? maximum‐likelihood regression with beta‐distributed dependent variables. Psychological Methods, 11, 54–71. Available from: 10.1037/1082-989x.11.1.54 16594767

[tpj71145-bib-0035] Sobhanian, H. , Song, W.‐Y. , Soltis, P.S. , Soltis, D.E. & Chen, S. (2026) Polyploidy and plant resilience to environmental stresses: molecular mechanisms and future applications. Plant Communications, 7, 101748. Available from: 10.1016/j.xplc.2026.101748 PMC13084105 41620822

[tpj71145-bib-0036] Steinegger, M. & Söding, J. (2017) MMseqs2 enables sensitive protein sequence searching for the analysis of massive data sets. Nature Biotechnology, 35, 1026–1028. Available from: 10.1038/nbt.3988 29035372

[tpj71145-bib-0037] The R Foundation . (2023) R: the R project for statistical computing . Available at: https://www.R‐project.org/

[tpj71145-bib-0038] Wang, T. , van Dijk, A.D.J. , Cai, X. , Wu, J. , Bonnema, G. & Wang, X. (2025) Brassica diversity through the lens of polyploidy: genomic evolution, introgression, and homoeologous exchange. Horticultural Plant Journal, 11, 1777–1790. Available from: 10.1016/j.hpj.2025.08.002

[tpj71145-bib-0039] Wickham, H. (2016) ggplot2: elegant graphics for data analysis. Available at: https://ggplot2.tidyverse.org

[tpj71145-bib-0100] Wood, S.N. (2017) Generalized additive models: an introduction with R, Second edition. New York: CRC Press. Available from: 10.1201/9781315370279

[tpj71145-bib-0040] Woolfenden, H. , Bilham, L. , O'Neill, C.M. , Penfield, S. , Irwin, J.A. , Morris, R.J. et al. (2026) A dataset of tissue‐specific gene expression during vegetative to floral development in Brassica napus . *FigShare*. Available at: 10.6084/m9.figshare.31325143

[tpj71145-bib-0041] Wu, J. , Lin, L. , Xu, M. , Chen, P. , Liu, D. , Sun, Q. et al. (2018) Homoeolog expression bias and expression level dominance in resynthesized allopolyploid *Brassica napus* . BMC Genomics, 19, 586. Available from: 10.1186/s12864-018-4966-5 PMC6080508 30081834

[tpj71145-bib-0042] Xiong, Z. , Gaeta, R.T. & Pires, J.C. (2011) Homoeologous shuffling and chromosome compensation maintain genome balance in resynthesized allopolyploid *Brassica napus* . Proceedings of the National Academy of Sciences, 108, 7908–7913. Available from: 10.1073/pnas.1014138108 PMC3093481 21512129

[tpj71145-bib-0043] Ziegler, D.J. , Khan, D. , Kalichuk, J.L. , Becker, M.G. , Pasha, A. , Provart, N.J. et al. (2025) Gene expression landscape of the *Brassica napus* seed reveals subgenome bias in both space and time. Plant Physiology, 198, kiaf283. Available from: 10.1093/plphys/kiaf283 PMC12305542 40580494

