# Supplementary material for: The degree and direction of subgenome expression bias in Brassica napus vary between cultivars, tissues and developmental stages
Source: Plant J. 2026 Oct 10;128(1):e71145. doi: 10.1111/tpj.71145 (PMC13653225; doi:10.1111/tpj.71145)
Supplement: Supplementary file 1 [file TPJ-128-0-s003.docx]

# Supplemental

*Table S1. List of homoeologue gene pairs. List of pairs of genes where the first gene is on the A subgenome and the second is on the C subgenome (see Materials and methods). The HGPs are within Darmor-bzh version 10 (Rousseau-Gueutin et al., 2020).*

*Table S2: List of significant Gene Ontology (GO) terms for the extremely biased homoeologue gene pairs (HGPs). The extremely biased HGPs are defined as those with their credible intervals within the top or bottom 5% of the distributions shown in Figure 5 (see Materials and methods).*

*Table S3. Counts of biased homoeologue gene pairs (HGPs) for each bias classification approach together with the number of HGPs in common. The numbers are used to test for significant bias in Figure S6.*

Figure S1. Total gene expression is biased to the C subgenome. The sum of the gene expression values (TPM) by subgenome in both tissues, and in genotypes, Stellar, Zhongshuang 11, and Ragged Jack. The start and end of the period of cold treatment are depicted by the vertical blue lines. Each point represents a sample. The solid line and its surrounding shaded region indicate the mean and credible interval from the Gaussian Process fit (see Materials and methods), with the colour indicating the subgenome (A: blue, C: red).

Figure S2. Mean expression of the genes shows that A subgenome dominates. The mean of the expression (TPM) for expressed genes by subgenome in both tissues, and in genotypes, Stellar, Zhongshuang 11, and Ragged Jack. The start and end of the period of cold treatment are depicted by the vertical blue lines. A gene is considered to be expressed if its TPM is above 1. Each point represents a sample with the shape of the point indicating the replicate. The solid line and its surrounding shaded region indicate the mean and credible interval from the Gaussian Process fit (see Materials and methods), with the colour indicating the subgenome (A: blue, C: red).

Figure S3: The relative expression for the HGPs of FLC is dynamic. The vertical axis shows Rel. A = A/(A+C), computed from the TPM values for the two genes in each HGP in each sample, across development, in both tissues, and in the indicated genotypes, Stellar, Zhongshuang 11, and Ragged Jack. There are 10 orthologs of FLC in B. napus (4 on the A subgenome and 6 on the C subgenome), which correspond to the three HGPs shown above. The apex and leaf data are shown in yellow and green, respectively. The start and end of the period of cold treatment are depicted by the blue vertical lines. Each point represents a sample. The solid line and its surrounding shaded region indicate the mean and credible interval from the Gaussian Process fit (see Materials and methods).

Figure S4: The relative expression for the HGPs of SOC1 is dynamic. The vertical axis shows Rel. A = A/(A+C), computed from the TPM values for the two genes in each HGP in each sample, across development, in both tissues, and in the indicated genotypes, Stellar, Zhongshuang 11, and Ragged Jack. There are 6 orthologs of SOC1 in B. napus (3 on the A subgenome and 3 on the C subgenome), which correspond to the three HGPs shown above. The apex and leaf data are shown in yellow and green, respectively. The start and end of the period of cold treatment are depicted by the blue vertical lines. Each point represents a sample. The solid line and its surrounding shaded region indicate the mean and credible interval from the Gaussian Process fit (see Materials and methods).

Figure S5. Comparison of subgenome bias classification methods to determine their impact on detecting bias. Distributions of the expression ratios for the homoeologue gene pairs (HGPs) of 14 day Stellar apex (replicate 1). The two expression filters are “A+C>2” (A, B and C) and “A>1 & C>1” (D, E and F). The three relative expression methods are “log A/C” (A, D), “pseudo log A/C” (B, E), and “Rel. A” (C, F). In each panel the total number of HGPs is given top-left with the number of HGPs that are considered to be not biased given top-right. The portion of the distribution that corresponds to the C-biased HGPs is to the left (red) with the number at the bottom-left, and the A-biased HGPs are to the right (blue) with the number shown at the bottom-right. The HGPs that fail to meet the criteria are considered unbiased (grey). The result of a $\chi^{2}$-test on the numbers of A- and C-biased HGPs is shown at the top-left. The fold-change cut-off (FCC) is 3 throughout (log_2_(3) = 1.58 to 2 d.p.), which is equivalent to cut-offs of 1/4 and 3/4 for “Rel. A” (C, F). See Materials and methods for full description of these expression filters and relative expression measures. The classification methods produce different numbers of biased HGPs in all cases except between (D) and (F). However, the methods producing the classification for (D) and (F) are equivalent for the expression filter and so the number of HGPs are expected to be the same. The number of C-biased HGPs is significantly higher than the number of A-biased HGPs in all cases except one (B) where not only are there more A-biased HGPs but the statistical test produces a not significant result.

Figure S6. Assessing the effect of different methods of HGP bias classification across the apex time-course in Stellar. The relative number of biased HGPs (A/(A+C)) (vertical axis) versus the time-point with each point representing a sample in the time-course. Bias was assigned to a HGP by a three step process: (1) filter the HGPs based on expression (“A+C>2” or “A>1 & C>1”), (2) calculate the relative expression (“log A/C”, “pseudo log A/C” or “Rel. A”), and finally, (3) label HGP as A-biased or C-based if the relative expression exceeds the fold-change cutoff (FCC) (see Materials and methods). The numbers of HGPs that are labelled as biased to each subgenome were then tested ($\chi^{2}$-test) with the significance indicated by the shape of the point (not significant (ns), <0.05 (*), <0.01 (**), <0.001 (***)). The numbers of biased HGPs showed that, in most of the methods, more HGPs were biased to the C subgenome than to A. A different fold-change cut-off (FCC) could result in samples that were significantly biased to C for a low FCC either losing the significance for a higher FCC, or even reversing the result so that some samples were significantly biased to A. This reversal could also happen when we changed either the initial expression filter or the bias quantification method. This analysis highlights the possibility of different HGP bias classification methods producing different overall bias inferences. The numbers used herein, together with the number of common HGPs, are given in Table S3.

Figure S7. Number of homoeologue gene pairs (HGPs) between pairs of chromosomes on the two subgenomes. The results are from the “mmseqs2 easy-rbh” pipeline after taking synteny into account using GENESPACE (see Materials and methods). The chromosomes for A and C are shown on the horizontal and vertical axes, respectively.

*Figure S8. The mean expression per gene depends on whether the genes are part of a HGP, or not. These plots show the relative mean expression, Rel. A = A/(A+C) with A and C referring to the mean of the TPMs for the expressed genes in the respective subgenomes, for each subgenome across development, in both tissues, and in the indicated cultivars, Stellar, Zhongshuang 11, and Ragged Jack. The apex and leaf data are shown in yellow and green, respectively. The start and end of the period of cold treatment are depicted by the blue vertical lines. Each point represents a sample. The solid line and its surrounding shaded region indicate the point-wise mean and credible interval from the Gaussian Process fit (see Materials and methods).*
